# Factors Influencing Individuals’ Intentions to Misuse LLM-Based Generative AI in Video Content Creation

**DOI:** 10.3390/bs16101816

**Published:** 2026-10-04

**Authors:** Ryeong Oh, Seongcheol Kim

**Affiliations:** 1 School of Communication and Media, Sookmyung Women’s University, Seoul 04310, Republic of Korea; reeoh@sm.ac.kr; 2 School of Media and Communication, Korea University, Seoul 02841, Republic of Korea

**Keywords:** generative AI, LLM, AI misuse, AI resistance, risk perception

## Abstract

Misusing generative AI in video content creation, such as creating deepfakes, leads to significant social losses and even crime. While individual users can become easily involved in generating social losses and committing crimes, scant research examines the factors influencing their intention to misuse generative AI in video content creation. To fill this gap, this study focuses on LLM-based GAI capable of manipulating linguistic information in videos, aiming to identify the key factors influencing individuals’ intentions to misuse generative AI, as well as their resistance to such technology. An online survey was conducted in June 2023, and data were collected from 400 respondents in Korea. The analyses identified associations of perceived misuse risk with misuse intention for AI voice and of favorability with resistance for AI human. Additionally, the mediation analyses identified significant indirect associations involving attitude and subjective norm in the relationships among risk perception, favorability, misuse intention, and resistance. These findings suggest that perceived misuse risk and attitudes toward disinformation creation are relevant correlates of misuse intention, rather than establishing that changing these perceptions would reduce misuse intention. Notably, the observed associations showed different descriptive patterns across media modalities. Furthermore, it highlights the relevance of perceived misuse risk when examining intentions to misuse LLM-based GAI.

## 1. Introduction

Significant progress in generative artificial intelligence (GAI) is anticipated to augment traditional video content creation processes. Video creation is divided into three stages: pre-production, production, and post-production. Among these, the production phase involves implementing various video elements, including voices, moving images, captions, and characters. Since GAI can automate the creation of video elements, it is expected to enhance efficiency by reducing the costs and time required in the production stages (Singh et al., 2023). GAI technologies can benefit, therefore, not only professional producers but also individual creators, enabling even those without professional expertise to create videos more conveniently.

However, although GAI remains in the early stages of adoption in the field of video content creation, its growing misuse in this domain is a matter of increasing concern. One primary way this technology is misused is by producing disinformation such as deepfakes and manipulated videos. Based on the OECD AI Incidents Monitor (AIM) data, the number of video-related AI incidents and hazards has increased in recent years. Reported deepfake-related incidents remained at approximately 30 cases annually until 2022 but increased dramatically to 212 in 2023, 491 in 2024, and 544 in 2025, reflecting the rapid escalation of deepfake misuse (OECD, 2026).

Another significant issue is that individuals can become key actors in generating disinformation through GAI. This technology greatly lowers the barriers individuals face when creating videos, making it easier for them to showcase their creativity through video content. Nevertheless, the simplicity of utilizing video content creation GAI also risks exploiting it to generate disinformation for amusement or financial gain. In the United States, a significant portion of the male population, approximately 20% of the 1500 respondents participating in this study, expressed an interest in acquiring the skills needed to produce deepfake pornography (Security Heroes, 2023). Furthermore, an alarming 1 in 10 individuals admitted to having already attempted creating deepfake porn involving celebrities or acquaintances. Worryingly, there is also a trend of deepfake creators being younger; many of such persons apprehended in South Korea over a five-month period were teenagers (The Korea Times, 2021). This suggests that misusing GAI is no longer limited to experts.

Image manipulation has thus far been the primary focus of concerns about deepfakes. Nevertheless, it is imperative to exercise caution when dealing with deepfakes that manipulate linguistic information. Modifying the components responsible for conveying linguistic information (e.g., voice and captions) in videos can more effectively transmit disinformation through visual and auditorial channels. If the generation technology is insufficient, image manipulation may disclose noticeable inconsistencies. However, linguistic manipulation is more difficult to detect and frequently more sophisticated and realistic than image manipulation. What is more, GAI can learn from existing data to rapidly generate linguistic media modalities such as audio, captions, and presenters. By combining such modalities, disinformation videos can be created. Indeed, disinformation videos that manipulate linguistic information have already been produced, causing societal confusion and disruption (BBC, 2024). In February 2024, a video was created in Hong Kong using a cleverly combined generated fake voice with images, impersonating a company official and giving detailed instructions to financial officers; this led to a loss of $25 million for that company (Milmo, 2024). In 2023, a video circulated on social media, purportedly showing footage of bombings in Gaza and shocking viewers with scenes of devastation; this was later revealed to be disinformation, a combination of old video footage with fake captions labeling the site as Gaza (Bond, 2023).

Specifically, a variety of GAIs based on quickly evolving large language models (LLMs) can produce disinformation that manipulates language data much more swiftly. LLMs not only generate text but also produce images and videos. LLM-based GAIs, which handle language more flexibly, are making it easier to create more refined voices, captions, and human-like figures, even if existing GAIs also generate images and text based on deep learning. With the rapid advancement of GAI in video content creation, disinformation can adopt many forms and manipulate language and visual modalities without restriction. Despite the current social issues arising from misusing GAI in video content creation, such as generating deepfakes, committing crimes, and invading others’ privacy, the primary focus of GAI-related video research remains on developing detection technologies. Such a dearth of research is untenable: a significant rise in the misuse of LLM-based GAI could lead to a surge in disinformation, causing information disorder and posing a threat to AI safety. A recent study raised concerns that partial synthesis could compromise cybersecurity, infringe on privacy, erode public trust, and ultimately undermine confidence in AI technologies (Zhou et al., 2025).

Moreover, immediately tackling such problems associated with creating disinformation becomes increasingly crucial with the growing distribution of LLM-based GAI for video content creation among individual users. Recent research has further emphasized the dual role of GenAI in the contemporary disinformation ecosystem. Agarwal et al. (2026) synthesized research on GenAI-enabled disinformation and proposed a framework that considers the motivational drivers and technological affordances involved in generating, manipulating, and disseminating misleading content. Their findings reinforce the importance of examining not only the technical capabilities of GAI but also the individual-level factors associated with its potential misuse.

Additionally, concerns about the potential dangers and misuse of GAI could hinder their acceptance, which may not be a desirable outcome. Resistance to change can hinder the adoption of new technologies and inhibit the progress of technological innovation (Ellen et al., 1991). Therefore, it is also essential to consider potential resistances arising from perceptions of LLM-based GAI misuse.

However, research aimed at identifying the underlying factors that cause GAI’s negative side effects remains insufficient. This study sheds light on this critical area by adopting a risk behavior perspective and making a significant attempt to shift the focus of LLM-based GAI misuse away from organizations and onto individual actors. The research goal is to uncover the emotional and cognitive factors influencing people’s misuse of LLM-based GAI technology, with a focus on disinformation perception. Additionally, this study reflects the characteristics of video content creation as a multimodal medium and places attention on language-based modality generation technologies that are particularly useful for information manipulation. We anticipate three main outcomes from this study. The first is gaining insights into how individuals perceive the risks and misuse of LLM-based GAI. The second expected outcome is to articulate a deeper understanding of how people perceive and react to LLM-based GAI systems that generate voices, captions, and presenters, as well as explain the media modalities most likely to influence misuse and resistance to LLM-based GAI. The third anticipated result is that the analysis will predict the likelihood of LLM-based GAI being misused and offer guidelines to safeguard society against such abuse.

This paper is organized as follows: Section 1 will examine the applicable areas of LLM-based GAI within video content creation. The next segment will explore the importance of investigating the inhibitory factors for adopting new technologies. Finally, Section 3 will present the research model and findings in detail, focusing on the variables that can be analyzed within a risk perception framework.

## 2. Literature Review and Research Hypotheses

### 2.1. Overview of LLM-Based Generative AI with Video Content Creation

Generative adversarial networks (GANs) are now the most widely utilized neural network architecture for producing video output using generative AI. Because a video is a collection of images, GANs analyze and synthesize pre-existing frames. While GANs can generate static images such as specific scenes or faces and enhance image resolution (Wen et al., 2018), their inability to incorporate motion and speed beyond given frames limits their application (Mehmood et al., 2022).

However, video is a multimodal medium, reflecting the producer’s intention and creativity through various combinations of modalities. Different elements and arrangements depend on both the video content and the creator’s skills. Videos combine audio and visual channels with language and non-language modalities (Bateman et al., 2017). The visual channel consists of numerous elements such as text and images, whereas the audio channel comprises various sounds, including voices and music (Zettl, 2012). Producers typically employ a variety of modalities to produce videos consistent with their vision. Using GAI at the production stage can make it easier to control the combination of modalities. Therefore, it is necessary to prioritize GAI, which can generate images and other modalities. Recent surveys on generative AI for film and video content creation likewise emphasize that text-driven systems are becoming increasingly capable of generating and editing multimodal content, ranging from scripted scenes to voiceovers and composited shots (Zhang et al., 2025).

These developments make LLM-based GAI particularly worthy of consideration.

Recently, people have started using LLM-based GAI for voice, caption, and digital human generation, advancing in a direction that enhances the development of modalities necessary for video content creation. The main advantage of LLM-based GAI systems is their superior ability to interpret complex language input. One notable service is Runway’s Gen-3, which generates high-quality results through inputting a simple text prompt. However, GAI-based video generation still faces challenges in maintaining realism and representing complex and dynamic movements. Although it may be relatively straightforward to reproduce specific facial expressions or character poses, limitations remain in generating fine-grained movements. Indeed, contemporary text-to-video systems often face challenges in maintaining spatial and temporal consistency, resulting in implausible object motion and discontinuities across frames (Yin et al., 2026). These limitations can make it difficult for creators to convey an effective or compelling narrative.

However, the linguistic modalities generated by LLM-based GAI have reached a highly sophisticated level of implementation in their output. These include expert voices, graphic typefaces, and presenters that closely resemble humans. Some services on the market provide AI-generated voices, captions, and presenters. Importantly, recent text-to-speech systems can generate high-quality, human-like speech and increasingly support expressive variation in speaking style, prosody, and emotion (Barakat et al., 2024). Notable services in this domain include Descript and Replica Studios, which offer advanced AI voice generators. AI captions analyze the input text or speech recognition from videos and offer a selection of elegantly designed fonts from which to choose. Adobe Premiere Pro (version 23.4), a well-known video editing software, provides an automatic captioning service that utilizes Adobe Sensei GAI. AI presenters can be created using advances in audio-driven talking-head generation, which enable the synthesis of human-like facial videos in which facial appearance and movements are generated or synchronized with speech (Rakesh et al., 2026). Companies like Synthesia and Deep Brain offer services for customizing these AI presenters to meet specific needs.

Recent advances in GAI have expanded the possibilities for integrated multimodal video production (Zhang et al., 2025). At the same time, AI voice, AI caption, and AI human presenter generation continue to develop as distinct production functions that users can select and combine according to their content creation needs. Rather than relying exclusively on fully automated video generation, individual creators can use these functions to produce or modify specific elements of video content. For example, voice-generation and captioning functions can be incorporated into social media video-editing workflows, allowing users to modify spoken or written information without generating an entirely new video. AI human presenters similarly enable the creation of human-like representations that deliver scripted information. These developments suggest that advances in multimodal GAI do not eliminate the relevance of examining individual modalities. Instead, the continued availability and selective use of these functions make it important to understand how users evaluate their distinct potential for misuse. Accordingly, the present study examines AI voice, AI caption, and AI human presenter generation as three modalities through which linguistic information in video content can be generated or manipulated.

Generally, highly skilled professionals are still required to ensure exceptional output in video content creation. Experienced presenters or reporters, well-versed in camera work and speaking with polished pronunciation, have managed the voice delivery while a team of experts crafted captions. AI-assisted editing tools can support novice creators in producing and refining video content by lowering technical barriers and enabling them to express and implement editing ideas more effectively (Tilekbay et al., 2024).

Nevertheless, with their user-friendly interfaces and high output quality, LLM-based GAI systems can also be used to disseminate false information. Manipulating linguistic information to imitate a celebrity’s voice or likeness, or providing captions that recontextualize otherwise unrelated footage, has become increasingly feasible. Recent evidence indicates that listeners may have difficulty distinguishing AI-generated voice clones from their real counterparts: participants perceived an AI-generated voice as having the same identity as its real counterpart approximately 80% of the time and correctly identified a voice as AI-generated only about 60% of the time (Barrington et al., 2025). Although AI-generated images can exhibit visual artifacts or artificiality, the use of cloned voices, contextualizing captions, and human-like presenters may enhance the perceived realism and plausibility of manipulated video content. As a result, LLM-based GAI may facilitate the dissemination of false information and contribute to societal confusion regarding the scale and apparent credibility of misinformation. Therefore, given the multiple components and modalities involved in video production, it is critical to prioritize research on the use of LLM-based GAI for disinformation creation.

### 2.2. Dual-Factor Structure of IT Usage

Research on new technologies emphasizes primarily positive outcomes such as adoption and utilization rates. However, introducing new technologies can sometimes result in negative outcomes, including misuse and resistance. It is thus essential to consider the latter, as they can thwart the positive functions of technology. When individuals engage in illegal, deviant, or unethical behavior that harms others or hinders performance, the potential benefits of adopting new technology can be undermined (Beaudry et al., 2020). In this context, the word ‘resistance’ refers to opposing a technology due to anticipated negative consequences, which hinders its acceptance and implementation (Bhattacherjee & Hikmet, 2007).

These negative outcomes can adversely affect society and reduce the positive acceptance of technology, necessitating the exploration of related factors. However, research on negative consequences has often been conducted using factors related to acceptance. For instance, previous studies have examined negative outcomes, assuming that a lack of factors contributing to acceptance would lead to the rejection of the technology. Nevertheless, undesirable results should not be equated with an absence of acceptance or usage. As demonstrated by Bhattacherjee and Hikmet (2007), resistance diminishes acceptance. However, it should be noted that simply reducing resistance does not automatically lead to increased acceptance. Furthermore, previous studies have suggested that different motivations drive negative outcomes and acceptance (Parthasarathy & Bhattacherjee, 1998; Venkatesh & Brown, 2001).

The dual-factor model of IT usage distinguishes the role of enablers in predicting acceptance and inhibitors in anticipating negative outcomes in IT use (Cenfetelli, 2004). Bhattacherjee and Hikmet (2007) argued that exploring the determinants of negative outcomes, distinct from acceptance-related concepts, is crucial. They suggest that misuse and resistance should not merely be seen as signs of non-adoption. Therefore, a closer examination of inhibitor factors is necessary to understand the process that leads to negative outcomes.

In the realm of video content creation, despite the increasing potential drawbacks of generative AI, important discussions have not been held regarding such technology’s acceptance and its accompanying risks. The proliferation of generative AI and problems related to disinformation has impacted various stakeholders and countries and has even victimized individuals. As awareness of generative AI issues as potential social and personal risks grows, researchers must prioritize examining factors that inhibit these hazards from a risk perspective.

The perception of risk is closely tied to the concept of resistance. Individuals often resist change when they perceive it as a potential threat to the status quo (Bhattacherjee & Hikmet, 2007). Resistance to adopting new technology can stem from various factors such as fear, high costs, a lack of skills, and concerns about privacy. One can therefore productively analyze misuse in terms of risk, given that misusing GAI leads to economic costs and privacy violations.

### 2.3. Perceived Risk Framework

The perceived risk framework provides a more comprehensive understanding of inhibitor factors from a risk perspective. This approach explores the intricate relationship between, on the one hand, subjective assessments of risk, emotional reactions, and cognitive perceptions and, on the other hand, how these elements influence decision-making behavior. Research that utilizes the perceived risk framework is primarily conducted to gain insights into individuals’ risk perceptions and their subsequent decision-making. It is also commonly employed in the field of technology acceptance to evaluate potential risks associated with technology.

#### 2.3.1. Perceived Risk to Technology

Perceived risk is a cognitive factor that has been recognized as a significant inhibitor to accepting technology. Notably, acceptance can be significantly affected by a perception of high risk (Hubert et al., 2019; Pavlou, 2003). Perceived risk among users can differ, encompassing economic, personal, and privacy concerns (Pavlou, 2003). In addition, the presence of social issues contributes to perceived risk (Cambefort & Roux, 2019).

In the context of GAI, the potential use of these technologies to create disinformation represents a further dimension of perceived risk. Recent research suggests that users’ evaluations of AI technologies involve both perceived risks and perceived benefits. Drawing on a cross-national survey of seven European countries, Hynek et al. (2025) showed that public perceptions of deepfake technologies vary across risk and benefit scenarios, including disinformation-related harms and legitimate creative applications. These findings underscore the need to examine perceived misuse risk as a distinct factor in users’ evaluations of GAI technologies, separately from their overall favorability toward the technology.

Uncertainty is crucial in shaping one’s perception of risk (Pavlou, 2003). Uncertainty surrounding technology arises when the results of utilizing it are difficult to predict or when the circumstances are online. A person’s perception of risk can be seen as beliefs about potential loss in uncertain situations. Thus, the concept of perceived risk has been widely seen as a crucial factor influencing the intention to engage in risky behavior and the inclination to resist it. One of the risky behaviors in digital environments is misuse, which is more likely to occur depending on the perceived risk. Research on misuse in the context of piracy has shown that individuals’ perceptions of legal risk can either decrease or increase such behaviors. In contrast, a higher perception of technical risk consistently leads to decreased risky behavior (Vida et al., 2012).

Resistance can arise in uncertain situations when individuals perceive threats to the status quo (Bhattacherjee & Hikmet, 2007). GAI in video content creation is a relatively new concept for many individuals, leading to unpredictable outcomes. As a result, this technology has a notable degree of uncertainty. The potential effects of misusing information, such as spreading false information, can be challenging to predict. Thus, analyzing how individuals perceive the risks associated with such technology becomes all the more imperative, as this crucially shapes their unfavorable attitudes toward generative AI.

Therefore, an individual’s level of uncertainty or confidence in GAI is often closely related to their perception of risk. There is a correlation between misuse and perceiving higher technological risk. The higher the perceived risk, the less likely people are to engage in misuse behaviors. Given the focus of this study, namely, risk perceptions toward GAI, it is anticipated that a heightened risk perception will result in decreased misuse. Additionally, since a higher perception of risk is known to be correlated with more resistance, it is expected that as the perception of the misuse risk of AI increases, resistance to such technology will also grow.

This study aims to analyze the cognitive and emotional assessment of LLM-based GAI, by considering the characteristics of video content creation methods and creators’ diverse preferences. Video creators use modalities such as voice, captions, and human presenters to convey specific information effectively through language. Furthermore, with services like AI voice, AI captions, and AI humans (presenters), LLM-based GAI significantly progressed toward expert-level precision, in contrast to image-based GAI, which is still developing. Accordingly, this study focuses on AI voice, AI caption, and AI human presenter generation as distinct modalities that enable individual creators to generate or manipulate linguistic information in video content. These technologies share LLM-based capabilities but differ in their modalities: auditory (voice), visual (caption), and audiovisual (human presenter). In the present study, perceived risk refers specifically to users’ perceptions of the potential for these technologies to be misused in creating disinformation. The study examines the hypothesized relationships within each modality and describes the observed patterns across modalities without conducting formal statistical tests of between-modality differences. Based on this framework, the following hypotheses are proposed:

**H1.**  *For each modality, perceived risk associated with LLM-based GAI is negatively associated with intention to misuse LLM-based GAI.*

**H2.**  *For each modality, perceived risk associated with LLM-based GAI is positively associated with resistance to LLM-based GAI.*

#### 2.3.2. Favorability

It is important to consider emotional factors alongside cognitive factors. Emotions are crucial to decision-making and can strongly influence one’s intention to use a technology, as they interact closely with cognitive factors (Chin et al., 2003; Shiau et al., 2022). Therefore, it is noteworthy that, recently, within the field of Human–Computer Interaction (HCI), there has been a significant increase in the number of studies focusing on emotional factors (Shiau et al., 2022).

In contrast to risk perception, which is formed within a context of uncertainty, positive emotions tend to reinforce certainty (Q. G. Ma & Wang, 2009). Positive emotions significantly impact behavior because they stimulate heuristic processing by activating schemas that tap into our existing memory (Fiske & Taylor, 2012; Shiau et al., 2022). Put simply, when positive emotions are present, they reinforce existing memories and strengthen convictions, thereby influencing usage behavior. Thus, it is likewise well known that positive emotions significantly reduce resistance. Nonetheless, positive emotions can also stimulate technology misuse. Research has indicated that when individuals perceive themselves as positive deviants, their upbeat emotions toward technology can increase misuse (Turgeman-Goldschmidt, 2008; Udris, 2016).

When exploring GAI, it is intriguing to investigate the impact that positive emotions toward technology have on the potential adverse effects of misuse. Measuring positive emotions toward technology can be done by determining an individual’s favorability toward a specific technology. Since optimism reflects an individual’s subjective feelings, favorability toward GAI is also a crucial variable.

Users’ favorability toward GAI may be influenced by their past experiences or memories, which can then shape their behavior through heuristic processing. Individuals who evaluate a technology positively may be more inclined to use it and less inclined to resist it. Because misuse is also a form of technology use, favorability toward GAI may be associated with misuse intention. However, favorability toward a technology should be distinguished from attitude toward its misuse. According to the theory of planned behavior (TPB), attitude refers to an individual’s evaluation of performing a specific behavior rather than a general evaluation of an object (Ajzen, 1991).

In the present study, favorability refers to an individual’s general affective evaluation of a GAI technology, whereas TPB attitude refers specifically to the evaluation of using that technology to create disinformation. A video creator may therefore favor a GAI technology for its usefulness in content production while disapproving of its misuse. Although favorability toward GAI and attitude toward its misuse are conceptually distinct, greater favorability toward the technology may be associated with stronger misuse intention and lower resistance. This study examines these relationships separately for AI voice, AI caption, and AI human presenter generation. Accordingly, the following hypotheses are proposed

**H3.**  *For each modality, favorability toward LLM-based GAI is positively associated with intention to misuse LLM-based GAI.*

**H4.**  *For each modality, favorability toward LLM-based GAI is negatively associated with resistance to LLM-based GAI.*

#### 2.3.3. Theory of Planned Behavior for Misuse

The theory of planned behavior (TPB) can predict and explain human behavior within the context of information technology. Research conducted on technology acceptance papers over the past 30 years reveals that TPB is among the most commonly utilized theories related to behavioral predictors (Marikyan et al., 2023). According to TPB, behavioral intention is influenced by three factors which determine a person’s actual behavior: attitude, subjective norm, and perceived behavioral control. Each element relates to a motivational disposition that influences an individual’s intention to engage in a particular behavior (Hartmann, 2009).

Attitude is shaped by behavioral beliefs, which are thoughts about the potential outcomes of an action. When individuals perceive the result of a behavior as both likely and desirable, they tend to develop a positive attitude toward that conduct. Subjective norms are developed through normative beliefs, which involve understanding of others’ expectations and desiring to conform to them. Subjective norms can influence behavior when people receive support and encouragement from those who are important to them, such as friends and parents. Perceived behavioral control is determined by one’s beliefs regarding the factors enabling or hindering a particular behavior. If individuals believe that they can navigate and overcome various circumstances that either support or impede their actions, they exhibit a high level of perceived behavioral control.

Research suggests that decisions involving uncertain outcomes and risks are often carefully considered (Fazio & Towles-Schwen, 1999; Esser, 1996; Payne et al., 1993; Hartmann, 2009). The TPB provides a framework for examining the cognitive factors associated with intentions to engage in risky behavior, including the misuse of GAI to create disinformation.

The TPB identifies attitude, subjective norm, and perceived behavioral control as key components associated with behavioral intention (Ajzen, 1991). In the context of GAI misuse, perceived risk and favorability toward the technology may be associated with these components, although they represent distinct evaluations of the technology.

Perceived risk concerns users’ recognition that GAI technologies may be used to create disinformation. Greater perceived risk may be associated with a less favorable attitude toward using these technologies for that purpose and less permissive subjective norms regarding such behavior. Perceived risk may also be associated with perceived behavioral control over disinformation creation, as both involve users’ assessments of the technology’s potential for misuse, although they represent distinct judgments about the technology and one’s own ability to perform the behavior.

Favorability toward GAI, by contrast, reflects a general positive evaluation of the technology rather than approval of its misuse. Favorable evaluations may be associated with a more positive attitude toward misuse, more permissive subjective norms, and greater perceived behavioral control when users feel more comfortable or confident using the technology. However, general favorability toward GAI does not necessarily imply the approval of disinformation creation or confidence in one’s ability to engage in such behavior.

Accordingly, this study examines how perceived risk and favorability toward LLM-based GAI are associated with attitude, subjective norm, and perceived behavioral control regarding GAI misuse. The following hypotheses are proposed:

**H5.**  *Perceived risk associated with LLM-based GAI is negatively associated with attitude, subjective norm, and perceived behavioral control regarding its misuse.*

**H6.**  *Favorability toward LLM-based GAI is positively associated with attitude, subjective norm, and perceived behavioral control regarding its misuse.*

Although the TPB primarily explains behavioral intentions, prior information technology research has extended its application to individuals’ responses to technological change. Kim and Kankanhalli (2009) examined self-efficacy for change in a model of user resistance to information systems implementation, identifying an indirect relationship through switching costs. Kwahk and Lee (2008) integrated the TPB and the technology acceptance model to examine readiness for change and technology use in ERP implementation. These studies provide a basis for considering perceived capabilities and control-related beliefs in examining responses to technology beyond behavioral intention.

The dual-factor perspective further suggests that factors encouraging technology use and those inhibiting it may coexist rather than represent opposite ends of a single continuum (Cenfetelli, 2004). Similarly, Sanford and Oh (2010) examined user resistance alongside factors associated with mobile data service adoption. Building on this literature, the present study treats misuse intention and resistance to GAI as distinct outcomes. Misuse intention refers to the intention to use GAI to create disinformation, whereas resistance refers to opposition to the technology more generally. Thus, disapproval of disinformation creation does not necessarily imply resistance to legitimate uses of GAI.

Nevertheless, evaluations of disinformation creation may be associated with broader responses to the technology. Accordingly, this study examines attitude, subjective norm, and perceived behavioral control regarding GAI misuse as separate mediators of the relationships between perceived risk and favorability on the one hand, and misuse intention and resistance on the other. The proposed indirect relationships extend the application of the TPB components to resistance without treating resistance as an established outcome of the TPB.

**H7.**  *Perceived risk associated with LLM-based GAI is indirectly associated with misuse intention through attitude, subjective norm, and perceived behavioral control regarding its misuse.*

**H8.**  *Perceived risk associated with LLM-based GAI is indirectly associated with resistance to the technology through attitude, subjective norm, and perceived behavioral control regarding its misuse.*

**H9.**  *Favorability toward LLM-based GAI is indirectly associated with misuse intention through attitude, subjective norm, and perceived behavioral control regarding its misuse.*

**H10.**  *Favorability toward LLM-based GAI is indirectly associated with resistance to the technology through attitude, subjective norm, and perceived behavioral control regarding its misuse.*

**Figure 1:**
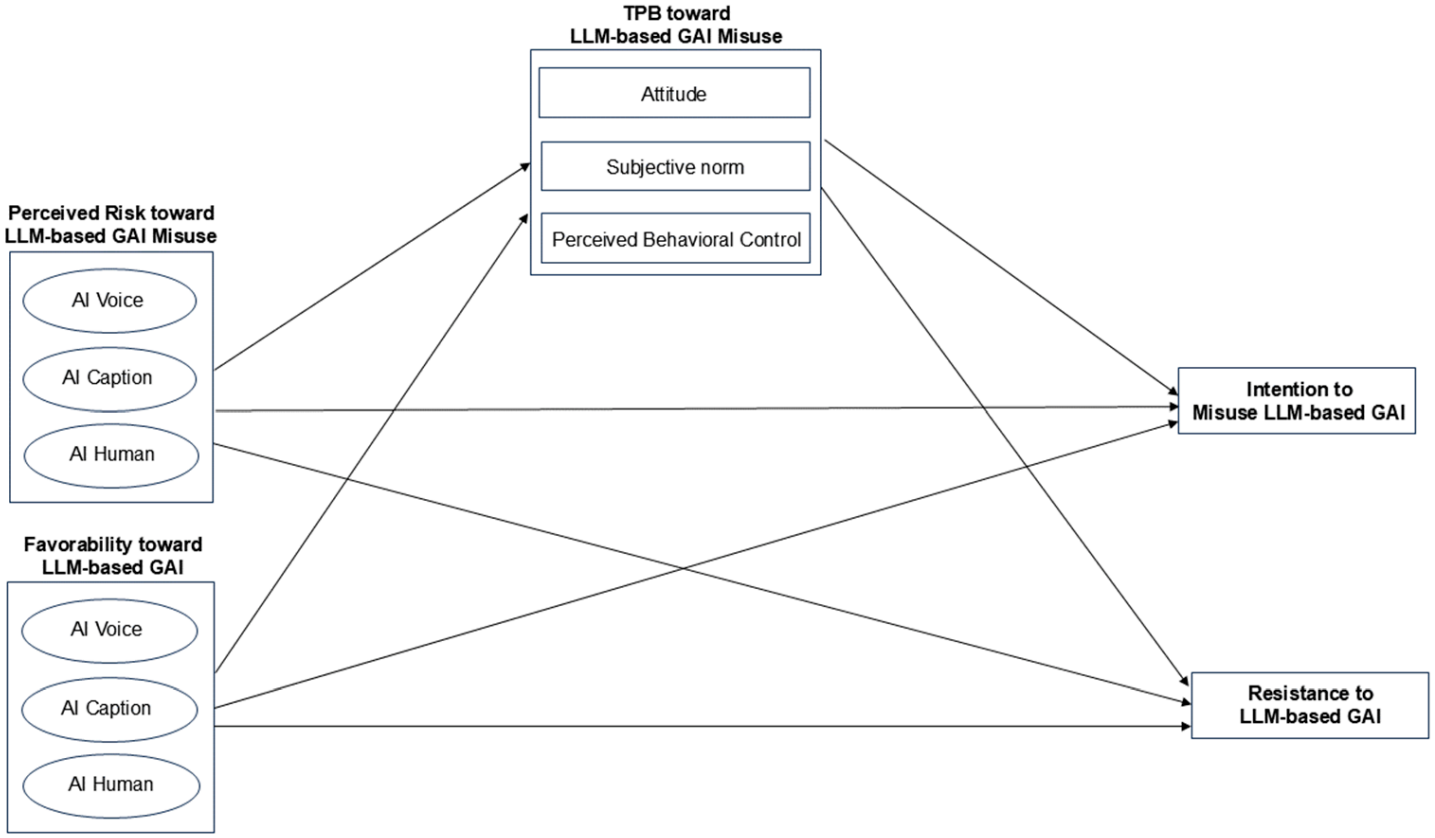
Conceptual research model.

The research model depicting this discussion is illustrated in Figure 1.

## 3. Methodology

### 3.1. Data Collection and Procedures

This study employed a quantitative methodology by administering a survey. The online survey was conducted from 8 June to 14 June 2023, using a panel provided by Surveylink, a survey company based in South Korea. To encompass a broader range of individuals, we included participants in their 20s to 50s rather than restricting the sample to younger age groups. Participants were required to have prior experience in creating and uploading video content. This criterion was intended to ensure experiential familiarity with video creation, rather than to selectively recruit individuals with high levels of technological knowledge or technology adoption. Such experience provided a relevant context for evaluating the potential use and misuse of GAI-based production tools. Accordingly, the sampling approach was aligned with the study’s objective of examining perceived risk, favorability, misuse intention, and resistance among individuals with video-creation experience, rather than representing the general population. The survey targeted adults aged 20 to 59 and employed quota sampling by gender and age. Recruitment for each gender and age cohort was closed once its allocated quota was reached. The final sample consisted of 200 men and 200 women. Respondents’ demographic profiles are detailed in Table 1.

Attendees were provided with a comprehensive overview of AI voice, AI caption, and AI human generation technologies, along with explanations of their practical applications. They then watched video content generated through LLM-based GAI and completed the survey. This study utilized AI generator services available in Korea as of June 2023, accessible to professionals and amateurs. ‘CLOVA Voice’ by NAVER was used for AI voice generation; ‘Vrew,’ developed by VoyagerX, was employed for AI caption generation; and ‘AI Studio,’ created by Deep Brain, was chosen for AI human generation. All three services are based on LLM generative models. CLOVA Voice utilizes Naver’s Korean LLM model, while Vrew and Deep Brain use GPT models. In addition, all three have been available since the early stages of GAI adoption in Korea. With their vast array of training data, these models provide creators with many options and generate outputs that align with the specific characteristics of video content creation explored in this paper. Thus, they were considered appropriate for this study.

### 3.2. Measures

The instrument items were based on prior research conducted on technology and adjusted to suit the current study’s specific needs. All items were measured using a 5-point scale.

In this study, perceived risk was operationalized narrowly as perceived misuse risk for disinformation creation, rather than as a multidimensional assessment of broader GAI-related risks. Specifically, it referred to the perceived risk that each GAI modality (AI voice, AI caption, and AI human) could be used to create disinformation. Favorability referred to an overall favorable evaluation of each GAI modality. Because the study aimed to examine modality-specific evaluations rather than to develop multidimensional latent scales of perceived risk and favorability, these constructs were measured separately for each modality using parallel single items.

TPB toward LLM-based GAI misuse was measured through attitude, subjective norms, and perceived behavioral control. Attitude is operationally defined as ‘attitude toward disinformation production using LLM-based GAI’; subjective norm as the ‘perception of people around you about using LLM-based GAI to create disinformation’; and perceived behavioral control as ‘perceived control over one’s skills in creating disinformation using LLM-based GAI.’ Items from Ajzen (2002) were adapted to measure LLM-based GAI misuse behavior.

Intention to misuse LLM-based GAI is operationally defined as respondents’ stated intention to use LLM-based GAI to create disinformation in the future, rather than their actual misuse behavior. Items from Taylor and Todd (1995) were adapted to assess prospective intentions to engage in disinformation creation using LLM-based GAI. Resistance to LLM-based GAI is operationally defined as ‘resistance to LLM-based GAI.’ The measurement items were adapted from Zaltman and Wallendorf (1979) and subsequent studies.

As shown in Table 2, reliability and validity were evaluated indirectly based on the communalities values obtained from the factor analysis of all items (Slocum-Gori & Zumbo, 2011). These values indicate the extent to which the items are explained by the factor structure; all items had values above 0.4, indicating their suitability. The items measured by the composite scales showed adequate reliability according to Cronbach’s alpha: TPB-attitude (3 items; α = 0.92), TPB-subjective norm (3 items; α = 0.90), TPB-perceived behavioral control (4 items; α = 0.82), intention to misuse LLM-based GAI (2 items; α = 0.86), and resistance to LLM-based GAI (3 items; α = 0.85). Furthermore, Barrett’s test indicated that each scale’s internal validity was satisfactory on a per item basis: TPB-attitude (*x*^2^ = 878.61, *p* < 0.001, KMO = 0.76), TPB-subjective norm (*x*^2^ = 753.70, *p* < 0.001, KMO = 0.75), TPB-perceived behavioral control (*x*^2^ = 597.00, *p* < 0.001, KMO = 0.77), intention to misuse LLM-based GAI (*x*^2^ = 341.72, *p* < 0.001, KMO = 0.50), and resistance to LLM-based GAI (*x*^2^ = 515.38, *p* < 0.001, KMO = 0.72).

### 3.3. Data Analysis

The SPSS 22 software package was used for data processing and coding. To examine the proposed hypotheses, this study utilized the PROCESS macro for SPSS and SAS (version 4.2 beta), a tool that has gained popularity among researchers studying mediation (Hayes & Rockwood, 2017). As Hayes (2013) explained, Model 4 of the PROCESS macro is designed to focus on simple mediation analysis. This model tests the hypothesis that the relationship between an independent variable (X) and a dependent variable (Y) is influenced by a third variable, referred to as the mediator (M). In the present study, PROCESS Model 4 was used to examine attitude, subjective norm, and perceived behavioral control as separate mediators of the relationships between perceived risk or favorability and misuse intention or resistance within each GAI modality.

Prior to the regression analyses, diagnostic checks were conducted to assess multicollinearity, normality of residuals, and homoscedasticity. Multicollinearity was evaluated using tolerance and variance inflation factor (VIF) values. In the full regression models, the minimum tolerance value was 0.277 and the maximum VIF was 3.610, indicating no serious multicollinearity concerns. The standardized residual histograms and normal P–P plots indicated no substantial violations of normality. In addition, plots of standardized residuals against standardized predicted values showed no clear systematic patterns indicative of substantial heteroscedasticity.

The PROCESS macro uses bootstrapping to assess the indirect effect’s statistical significance. Bootstrapping is a method that entails repeatedly sampling from the data (with replacement) and estimating the indirect effect for each sample. This process helps researchers construct an empirical approximation of the sampling distribution of the indirect effect. Then, the confidence interval for the indirect effect is calculated based on these bootstrap samples. If the confidence interval does not include zero, it suggests that the indirect effect is statistically significant. As discussed by Celli (2022), mediation analysis enables the examination of the distinct direct and indirect effects of an independent variable on an outcome. In this study, 5000 bootstrap samples were used to estimate the indirect effects.

Age, gender, and production experience were included as covariates in all PROCESS Model 4 mediation analyses. To account for multiple comparisons, a Bonferroni correction was applied separately within each mediation hypothesis family (H7–H10). Each family involved nine indirect-effect tests (three modalities × three TPB mediators), yielding an adjusted significance threshold of 0.00556 (0.05/9) and 99.4444% bootstrap confidence intervals. An indirect association was considered statistically significant when its adjusted interval excluded zero.

Standardized regression coefficients (β) and R^2^ values were reported alongside unstandardized coefficients to aid the interpretation of the magnitude of the observed associations and model explanatory power. A sensitivity power analysis using G*Power (version 3.1.9.7) indicated that, with N = 400, 12 predictors, α = 0.05, and power = 0.80, the minimum detectable overall regression effect was f^2^ = 0.045; this analysis does not estimate power for the bootstrapped indirect effects.

Rather than evaluating all relationships among the variables in a single integrated structural model, this study examined the direct and specific indirect associations separately for AI voice, AI caption, and AI human presenter generation. This modality-specific approach allowed the study to identify which TPB components mediated the associations of perceived risk and favorability with misuse intention and resistance within each modality.

To assess potential common method bias, Harman’s single-factor test was conducted using all 21 measurement items. The unrotated principal component analysis extracted five factors with eigenvalues greater than 1.0, accounting for 69.10% of the total variance. The first factor accounted for 33.81% of the variance, followed by the second (10.94%), third (10.80%), fourth (7.00%), and fifth (6.55%) factors. Thus, the variance was distributed across multiple factors rather than being predominantly explained by a single factor, with the first factor accounting for less than 50% of the total variance. These results suggest that common method bias was unlikely to be a dominant concern in the present data.

## 4. Results

The correlations between the key variables and their means are presented in Table 3 and Table 4. Gender, age, and production experience were included as control variables in the analyses.

Table 5 presents the hypothesis test results. The perceived risk and favorability toward LLM-based GAI for each modality had distinct impacts on the intention to misuse the technology and resistance to it. Risk perception concerning AI voice, AI caption, and AI human statistically significantly affected the intention to misuse GAI. The lower the risk, the higher the intention to misuse. Conversely, the higher the risk perception regarding AI voice, the greater the resistance to GAI. Favorability toward AI human had a statistically significant effect on the intention to misuse LLM-based GAI, and favorability toward AI voice significantly affected resistance to the technology. Higher favorability concerning AI human increased misuse intentionality, whereas lower favorability regarding AI voice heightened resistance. Therefore, H1, H2, H3, and H4 were partially supported.

Next, we examined the role of TPB in the effects of perceived risk and favorability toward GAI on misuse and resistance. Initially, we analyzed the impact of perceived risk and favorability concerning LLM-based GAI on TPB components toward the technology. The analysis demonstrated that the effects of perceived risk and favorability toward GAI on TPB varied by modality. For example, perceived risk related to AI voice and AI human influenced attitude and subjective norm. Lower perceived risk associated with AI voice and AI human corresponded with higher attitude and subjective norm. As for favorability toward AI voice and AI caption, this affected perceived behavioral control; higher favorability toward these modalities increased perceived behavioral control. As for AI human, favorability influenced all TPB components, with higher favorability correlating with stronger TPB scores. Thus, H5 and H6 were partially supported.

**Figure 2:**
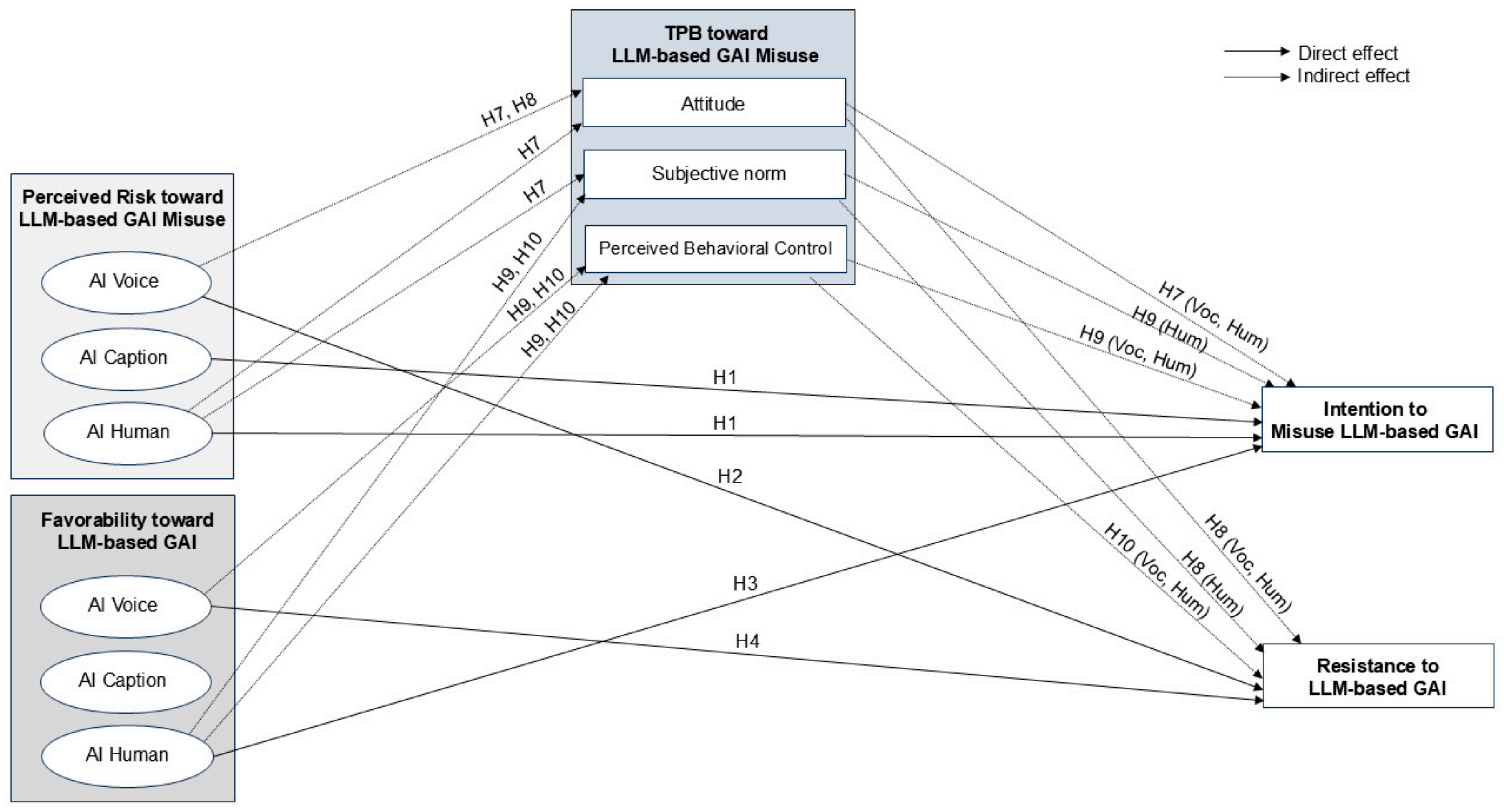
Results of hypothesis testing in the research model.

As shown in Table 6, the mediation analyses were evaluated using Bonferroni-adjusted 99.4444% bootstrap confidence intervals to account for the nine indirect-effect tests conducted within each hypothesis family. In examining the relationship between perceived risk toward LLM-based GAI and misuse intention, significant indirect associations were observed through attitude for AI voice and through both attitude and subjective norm for AI human. The same pattern emerged for resistance, with significant indirect effects through attitude for AI voice and through both attitude and subjective norm for AI human. Regarding favorability toward LLM-based GAI, significant indirect associations with misuse intention were observed through perceived behavioral control for AI voice and through subjective norm and perceived behavioral control for AI human. A similar pattern was found for resistance, with significant indirect effects through perceived behavioral control for AI voice and through subjective norm and perceived behavioral control for AI human. No significant indirect effects involving AI caption were observed after the multiple-comparison adjustment. Thus, H7, H8, H9, and H10 were partially supported. Figure 2 presents the significant direct and indirect paths.

## 5. Discussion and Conclusions

This study focused on GAI misuse in disinformation video content creation and analyzed factors contributing to the technology’s misuse and resistance to it. Given the uniqueness and complexity of video manipulation, the study restricted its consideration to LLM-based GAI, which generates linguistic information. This study mainly emphasized voice, caption, and human-like presenter generation using AI. Our paper examined how perceived misuse risk and favorability toward LLM-based GAI are associated with misuse intention and resistance, including indirect associations through attitude, subjective norm, and perceived behavioral control, the three components of the theory of planned behavior. Upon examination, the analysis generated the following discoveries.

Among the most interesting findings was that, for all modalities, lower perceived misuse risk was associated with greater intention to misuse LLM-based GAI. Contrary to the expectation that higher perceived risk would be associated with lower misuse intention, our findings highlighted the relevance of low perceived misuse risk. This finding should be considered in light of the video production context in which the present study was conducted. From a risk–benefit perspective, users’ evaluations of the potential benefits and risks associated with technology adoption are relevant to understanding their responses to technology (Tang et al., 2025; N. Ma et al., 2026). In the context of video production, the perceived benefits of GAI may include reduced production time, lower resource requirements, and greater efficiency in content creation and distribution. These production-related benefits may be particularly salient to individuals with prior experience in creating and uploading videos. The combination of salient production benefits and low perceived misuse risk may help explain why individuals who perceive less risk associated with the potential use of GAI to create disinformation report greater intentions to engage in such misuse. Thus, the present study suggests that the relationship between risk perception and misuse intention may depend on the context in which a technology is evaluated, rather than assuming that heightened risk perception uniformly discourages misuse.

Furthermore, several hypotheses in this study received partial support, likely due to the differences in each modality’s characteristics. The relationship among risk perception, favorability, and resistance toward LLM-based GAI was found to depend on the modality and TPB factors. When it comes to AI voice and AI human, the ways people perceive risk and favorability can have different impacts on their intentions to misuse or resist LLM-based GAI.

AI voice showed significant associations with resistance to LLM-based GAI. Both perceived risk and favorability toward AI voice were associated with resistance to the technology. Resistance exhibited a positive correlation with perceived risk concerning AI voice and a negative correlation with perceived favorability toward AI voice. These results suggest that negative experiences or memories related to voice manipulation may be associated with heightened risk perception and resistance. Voice attributes are less complex than visual modalities, easier to believe in, and can be easily generated with text input. In Korea, voice phishing has long been recognized as a serious social crime; such awareness may contribute to heightened awareness of voice deception and greater resistance. Therefore, users may have already encountered disinformation from voice manipulation. It can thus be inferred that uncertainty regarding risks associated with voice manipulation may be related to perceived risk and resistance to related technologies.

In contrast to voice, AI human showed significant associations between perceived misuse risk, favorability, and misuse intention. Higher favorability and lower perceived misuse risk toward AI human were associated with greater intentions to misuse GAI. The tendency for misuse intention to be higher, particularly when favorability concerning the technology is higher, highlights the potential for AI misuse. This is particularly relevant when individuals choose AI human to acquire knowledge about the technology and showcase their expertise. Put simply, AI human may be accepted, but it can also have the potential for misuse. While AI human’s impact has not yet been fully realized, it is important to scrutinize its potential for future misuse.

The findings for AI caption warrant further consideration. Although lower perceived misuse risk associated with AI caption was related to greater misuse intention, favorability toward AI caption was not significantly associated with misuse intention or resistance, and the caption-related specific indirect effects through the TPB components were not statistically significant after adjustment for multiple comparisons. This pattern may be interpreted in light of Sundar’s (2008) MAIN model, which suggests that digital media modalities provide cues that shape users’ evaluations. AI-generated captions may make human imitation less salient than the other two modalities, whereas AI voice and AI human presenters can reproduce recognizable human attributes, such as a person’s voice or appearance, making their potential use for impersonation or deception relatively apparent. This interpretation may help explain why perceived misuse risk associated with AI caption was related to misuse intention while showing more limited associations with resistance and the TPB-mediated pathways.

Furthermore, the mediation findings show different descriptive patterns of indirect associations through TPB components across AI voice and AI human; these patterns were not subjected to formal between-modality tests. From the perspective of deterrence theory, informal sanctions may include social disapproval, self-disapproval, and moral inhibition (Simpson, 2002; Pratt et al., 2006). Attitudes toward misuse and subjective norms may reflect aspects of these informal constraints, although they should not be regarded as direct measures of sanctions. In the present study, perceived misuse risk was indirectly associated with misuse intention through attitude for AI voice and through both attitude and subjective norm for AI human. Favorability, however, showed a different pattern: its indirect association with misuse intention operated through perceived behavioral control for AI voice and through subjective norm and perceived behavioral control for AI human. These findings suggest that the observed associations involving psychological and social factors may vary descriptively according to both the GAI modality and the type of technology evaluation. Accordingly, future research on approaches to discouraging misuse may consider users’ awareness of misuse-related risks, attitudes toward misuse, perceived social expectations, and perceived behavioral control, while testing whether the observed modality-specific patterns replicate.

In summary, these findings carry the following important implications. From a theoretical perspective, we suggest that distinguishing between linguistic and non-linguistic modalities is essential for conducting effective multimodal GAI research. Furthermore, the distinct associations of perceived risk and favorability underscore the value of examining both emotional and cognitive evaluations when studying correlates of misuse intention and resistance. Additionally, within the perceived risk framework, we identified indirect associations through TPB components, including attitude and subjective norm, without establishing that these components causally discourage LLM-based GAI misuse.

Next, we set our study apart from previous research in that we did not simply present users’ evaluations of the technology. Instead, we discovered that it is essential to address complacent perceptions of misuse by including TPB. The study also identifies practical implications for stakeholders involved in developing, using, and governing GAI-based video production.

The findings also suggest that practical interventions should address the distinct misuse risks associated with each GAI modality rather than rely solely on general awareness campaigns. Although lower perceived misuse risk was associated with greater misuse intention across all three modalities, the relationships involving favorability, resistance, and the TPB components varied by modality. Accordingly, educational initiatives, platform safeguards, and policy responses could be tailored to the specific ways in which AI voice, AI caption, and AI human may be used to create disinformation.

For AI voice, prevention efforts could focus on voice impersonation and voice-based deception, including phishing and the unauthorized replication of recognizable voices. Public education could use concrete examples of synthetic voice manipulation and emphasize that a familiar-sounding voice should not, by itself, be treated as proof of a speaker’s identity. Developers of voice-generation services could also consider consent-verification procedures for cloning identifiable voices and clear guidance against impersonation. These measures are particularly relevant given that perceived risk and favorability toward AI voice were associated with resistance in the present study.

For AI human, practical safeguards could address the deceptive use of synthetic presenters and the unauthorized reproduction of an identifiable person’s appearance or identity. Developers and video platforms could consider consent requirements for identity-based avatar creation, clear disclosure when realistic presenters are synthetically generated, and mechanisms for reporting impersonation or misleading representations. Public education could likewise illustrate how a realistic AI presenter may appear credible even when the statements attributed to that presenter are fabricated. These recommendations are relevant to the finding that lower perceived misuse risk and greater favorability toward AI human were associated with greater misuse intention.

For AI caption, interventions could focus on misinformation and contextual manipulation rather than identity impersonation. Media literacy programs could demonstrate how captions may alter the apparent meaning of an interview, speech, or other video footage, and encourage viewers to compare captions with the original audio and surrounding context when the accuracy of a claim matters. Video-editing and caption-generation services could also consider providing accessible ways to review caption changes and correct misleading text. Although the caption-related indirect effects were limited in the present study, this should not be interpreted as indicating that caption manipulation poses little risk. As discussed above, its potential misuse may simply be less salient to users than voice or human-presenter manipulation.

For policymakers, these modality-specific risks provide a basis for developing more concrete guidance for AI service providers and public education programs. Rather than communicating only that generative AI can be misused, such initiatives could illustrate recognizable scenarios of voice impersonation, identity-based avatar deception, and caption-driven contextual manipulation. At the same time, these measures should distinguish legitimate creative uses of GAI from deceptive uses, so that efforts to reduce misuse do not unnecessarily discourage beneficial video-production practices.

Several limitations should be considered when interpreting the findings. First, the study employed a cross-sectional survey design, which does not establish the temporal ordering or causal direction of the observed relationships. Accordingly, the significant indirect associations identified in the mediation analyses should not be interpreted as evidence of causal mediation. Moreover, the modality-specific PROCESS analyses did not assess the overall fit of the proposed relationships as an integrated structural model. Future longitudinal or experimental studies could clarify the temporal relationships, while structural equation modeling could evaluate the overall theoretical model. In addition, perceived misuse risk and favorability were each measured using a single item for each GAI modality. These measures captured respondents’ general recognition of the potential for disinformation creation and their overall favorability toward the technology, respectively. However, single-item measures do not capture the full range of possible dimensions within these constructs and do not permit an assessment of internal consistency. Future research could employ multi-item measures to examine different dimensions of perceived misuse risk and technology favorability more comprehensively. Self-reported misuse intention should also not be interpreted as evidence of actual misuse behavior.

Second, the study focused on South Korean adults aged 20–59 with prior experience in creating and uploading video content. This sampling criterion was intentionally adopted to examine individuals who could evaluate GAI technologies in relation to video-production activities. South Korea also provides a relevant context for examining AI caption generation because captions are extensively used in Korean video content, not only to transcribe speech but also to convey emotions, emphasize expressions, and provide contextual or entertaining information. This established production practice makes Korean video creators a suitable population for an initial examination of AI caption generation alongside AI voice and AI human presenters. Future research employing different sampling designs or cross-cultural comparisons could examine whether the observed relationships also apply to individuals with different levels of video-production experience, other demographic groups, or different video-production contexts.

Third, the data were collected in June 2023. Since then, GAI technologies for video production have advanced considerably, particularly in the quality and accuracy of generated outputs. Nevertheless, AI voice, AI caption, and AI human presenter generation continue to exist as distinct production functions, even as increasingly integrated video-generation technologies have emerged. Accordingly, examining these modalities separately remains relevant for understanding how users evaluate their potential misuse. Future research could investigate whether improvements in generation quality and the growing integration of these functions are associated with changes in perceived misuse risk, favorability, misuse intention, and resistance. This approach could also be extended to image generation and fully automated multimodal video production.

## Figures and Tables

**Table 1:** Demographic profile.

Measure	Items	Frequency	Percent
Gender	Male	200	50
	Female	200	50
Age	20–29	100	25
	30–39	100	25
	40–49	100	25
	50–59	100	25
Experience in creating video uploading	1–4 times	311	77.8
	5–9 times	34	8.5
	Over 10 times	55	13.8

**Table 2:** Measurement items.

Item	Measure	Communalities	Cronbach’s Alpha	KMO
PR Voice	There is a risk that AI voice could be used to create disinformation.	0.73		
PR Caption	There is a risk that AI caption could be used to create disinformation.	0.69		
PR Human	There is a risk that AI human could be used to create disinformation.	0.62		
Fav Voice	I favor AI Voice.	0.61		
Fav Caption	I favor AI Caption.	0.57		
Fav Human	I favor AI Human.	0.58		
Att 1	I think using these AI technologies to create disinformation is wise.	0.74	0.92	0.76
Att 2	I like using these AI technologies to create disinformation.	0.78		
Att3	I think using these AI technologies to create disinformation is positive.	0.76		
Norm 1	Someone important to me would want me to use these AI technologies to create disinformation.	0.72	0.90	0.75
Norm 2	Someone important to me would use these AI technologies to create disinformation.	0.77		
Norm 3	Someone important to me would encourage me to use these AI technologies to create disinformation.	0.76		
Control 1	I am confident in my ability to create disinformation using these AI technologies.	0.73	0.82	0.77
Control 2	I am skilled at creating disinformation using these AI technologies.	0.74		
Control 3	It would be possible to communicate with others using disinformation created with these AI technologies.	0.53		
Control 4	It would be easy for me to create disinformation using these AI technologies.	0.68		
Misuse 1	I will continue to use these AI technologies to create disinformation in the future.	0.60	0.86	0.50
Misuse 2	I will frequently use these AI technologies to create disinformation in the future.	0.64		
Resist 1	I am resistant to these AI technologies.	0.76	0.85	0.72
Resist 2	I am dissatisfied with these AI technologies.	0.77		
Resist 3	I have something to criticize about these AI technologies.	0.74		

**Table 3:** Correlations among key variables.

	1	2	3	4	5	6	7	8	9	10	11	12	13	14
1. Gender	1	0.000	−0.049	−0.022	0.040	0.051	0.006	−0.024	−0.133 **	−0.150 **	−0.168 **	−0.113 *	−0.144 **	−0.048
2. Age		1	−0.044	0.084	0.125 *	0.013	0.138 **	0.032	0.262 **	0.032	0.040	0.035	0.098 *	−0.088
3. Exp			1	−0.060	−0.125 *	0.036	0.056	0.090	0.059	−0.032	−0.066	0.049	−0.020	0.015
4. Risk Voc				1	0.498 **	0.520 **	−0.005	0.035	−0.068	−0.171 **	−0.110 *	0.022	−0.232 **	0.102 *
5. Risk Capt					1	0.345 **	−0.045	−0.010	0.004	−0.061	−0.026	−0.070	−0.107 *	0.007
6. Risk Hum						1	0.053	0.175 **	−0.129 **	−0.317 **	−0.291 **	−0.052	−0.350 **	−0.060
7. Fav Voc							1	0.354 **	0.391 **	−0.027	−0.009	0.168 **	0.087	−0.124 *
8. Fav Capt								1	0.254 **	−0.074	−0.075	0.138 **	0.014	0.027
9. Fav Hum									1	0.150 **	0.173 **	0.180 **	0.296 **	−0.025
10. Att										1	0.840 **	0.396 **	0.614 **	0.401 **
11. Norm											1	0.420 **	0.625 **	0.442 **
12. Ctrl												1	0.369 **	0.247 **
13. Misuse													1	0.184 **
14. Resist														1

Note: N = 400; * *p* < 0.05, ** *p* < 0.01.

**Table 4:** The means for each key variable.

			Risk Voc	Risk Capt	Risk Hum	Fav Voc	Fav Capt	Fav Hum	Att	Norm	Ctrl	Misuse	Resist
Total		M	3.44	3.20	3.72	3.73	3.81	3.46	2.28	2.40	3.08	2.66	2.84
		(SD)	0.90	0.94	1.14	0.81	0.84	1.04	1.17	1.11	0.85	1.20	0.92
Gender	Male	M	3.46	3.16	3.66	3.73	3.83	3.60	2.46	2.59	3.18	2.83	2.88
	(N = 200)	(SD)	0.91	0.96	1.20	0.83	0.88	1.05	1.16	1.10	0.88	1.18	0.95
	Female	M	3.42	3.24	3.78	3.74	3.79	3.33	2.11	2.22	2.99	2.49	2.80
	(N = 200)	(SD)	0.90	0.92	1.07	0.79	0.79	1.01	1.14	1.09	0.82	1.19	0.88
Age	20s	M	3.27	2.91	3.60	3.54	3.76	3.02	2.29	2.32	3.05	2.44	2.91
	(N = 100)	(SD)	0.91	0.90	1.26	0.94	0.91	1.13	1.15	1.02	0.88	1.19	0.87
	30s	M	3.55	3.28	3.87	3.70	3.81	3.44	2.10	2.37	3.06	2.66	2.97
	(N = 100)	(SD)	0.82	0.94	1.00	0.78	0.79	0.98	1.17	1.15	0.90	1.27	0.90
	40s	M	3.39	3.37	3.70	3.86	3.84	3.62	2.45	2.50	3.09	2.80	2.72
	(N = 100)	(SD)	0.94	0.87	1.11	0.80	0.86	1.00	1.17	1.11	0.79	1.11	0.91
	50s	M	3.55	3.23	3.70	3.82	3.83	3.77	2.28	2.41	3.13	2.74	2.76
	(N = 100)	(SD)	0.93	0.99	1.18	0.66	0.79	0.89	1.17	1.16	0.86	1.19	0.97
Exp	1~4 times	M	3.47	3.25	3.68	3.70	3.77	3.42	2.29	2.43	3.06	2.66	2.83
	(N = 311)	(SD)	0.86	0.90	1.12	0.76	0.84	1.00	1.14	1.09	0.84	1.16	0.89
	5~9 times	M	3.29	3.21	3.97	3.88	3.88	3.71	2.35	2.44	3.15	2.76	2.96
	(N = 34)	(SD)	0.97	1.15	1.11	0.98	0.73	1.17	1.21	1.20	0.98	1.25	0.93
	10+	M	3.35	2.89	3.75	3.80	3.98	3.55	2.16	2.20	3.17	2.56	2.84
	(N = 55)	(SD)	1.11	0.98	1.24	0.97	0.85	1.14	1.29	1.18	0.88	1.36	1.06

**Table 5:** Direct effect hypothesis testing results.

Hyp.	Path	B	*β*	R^2^	t-Value	*p*-Value	Result
**H1**	**PR → Misuse**						
a	Voc → Misuse	−0.327	−0.248	0.092	−5.138	0.000 **	**Supported**
b	Capt → Misuse	−0.152	−0.120	0.045	−2.396	0.017 *	**Supported**
c	Hum → Misuse	−0.362	−0.345	0.149	−7.416	0.000 **	**Supported**
**H2**	**PR → Resist**						
a	Voc → Resist	0.111	0.110	0.022	2.195	0.029 *	**Supported**
b	Capt → Resist	0.021	0.021	0.011	0.422	0.673	Rejected
c	Hum → Resist	−0.046	−0.057	0.013	−1.133	0.258	Rejected
**H3**	**Favor → Misuse**						
a	Voc → Misuse	0.114	0.077	0.037	1.549	0.122	Rejected
b	Capt → Misuse	0.013	0.009	0.031	0.185	0.853	Rejected
c	Hum → Misuse	0.320	0.277	0.101	5.545	0.000 **	**Supported**
**H4**	**Favor → Resist**						
a	Voc → Resist	−0.130	−0.115	0.023	−2.287	0.023 *	**Supported**
b	Capt → Resist	0.030	0.028	0.011	0.551	0.582	Rejected
c	Hum → Resist	−0.009	−0.010	0.010	−0.194	0.846	Rejected
**H5**	**PR → TPB**						
a	Voc → Att	−0.234	−0.181	0.057	−3.695	0.000 **	**Supported**
b	Voc → Norm	−0.151	−0.123	0.050	−2.495	0.013 *	**Supported**
c	Voc → Ctrl	0.018	0.019	0.016	0.376	0.707	Rejected
d	Capt → Att	−0.082	−0.066	0.029	−1.307	0.192	Rejected
e	Capt → Norm	−0.039	−0.034	0.036	−0.668	0.505	Rejected
f	Capt → Ctrl	−0.060	−0.066	0.020	−1.314	0.190	Rejected
g	Hum → Att	−0.317	−0.310	0.120	−6.554	0.000 **	**Supported**
h	Hum → Norm	−0.274	−0.282	0.114	−5.935	0.000 **	**Supported**
i	Hum → Ctrl	−0.037	−0.049	0.018	−0.976	0.330	Rejected
**H6**	**Favor → TPB**						
a	Voc → Att	−0.042	−0.029	0.026	−0.583	0.561	Rejected
b	Voc → Norm	−0.013	−0.010	0.035	−0.193	0.847	Rejected
c	Voc → Ctrl	0.174	0.165	0.043	3.312	0.001 **	**Supported**
d	Capt → Att	−0.106	−0.076	0.031	−1.535	0.126	Rejected
e	Capt → Norm	−0.099	−0.075	0.041	−1.509	0.132	Rejected
f	Capt → Ctrl	0.134	0.131	0.033	2.644	0.009 **	**Supported**
g	Hum → Att	0.154	0.137	0.042	2.646	0.009 **	**Supported**
h	Hum → Norm	0.170	0.159	0.058	3.100	0.002 **	**Supported**
i	Hum → Ctrl	0.138	0.168	0.042	3.251	0.001 **	**Supported**

Note: * *p* < 0.05, ** *p* < 0.01. Note: B = unstandardized coefficient; β = standardized coefficient; R^2^ = coefficient of determination. Age, gender, and production experience were included as covariates in the analyses.

**Table 6:** Indirect effect hypothesis testing results.

Hyp.	Path	*p*-Value	Effect	LLCI	ULCI	Result
**H7**	**PR → TPB → Misuse**					
a	Voc → Att → Misuse	0.000 **	−0.138	−0.260	−0.032	**Supported**
b	Voc → Norm → Misuse		−0.097	−0.229	0.019	Rejected
c	Voc → Ctrl → Misuse		0.009	−0.070	0.087	Rejected
d	Capt → Att → Misuse	0.017 *	−0.050	−0.163	0.060	Rejected
e	Capt → Norm → Misuse		−0.026	−0.144	0.087	Rejected
f	Capt → Ctrl → Misuse		−0.030	−0.109	0.045	Rejected
g	Hum → Att → Misuse	0.000 **	−0.178	−0.273	−0.102	**Supported**
h	Hum → Norm → Misuse		−0.167	−0.274	−0.077	**Supported**
i	Hum → Ctrl → Misuse		−0.017	−0.079	0.038	Rejected
**H8**	**PR → TPB → Resist**					
a	Voc → Att → Resist	0.029 *	−0.081	−0.157	−0.016	**Supported**
b	Voc → Norm → Resist		−0.059	−0.141	0.010	Rejected
c	Voc → Ctrl → Resist		0.005	−0.036	0.052	Rejected
d	Capt → Att → Resist	0.673	−0.026	−0.089	0.031	Rejected
e	Capt → Norm → Resist		−0.015	−0.081	0.050	Rejected
f	Capt → Ctrl → Resist		−0.016	−0.066	0.026	Rejected
g	Hum → Att → Resist	0.258	−0.108	−0.167	−0.059	**Supported**
h	Hum → Norm → Resist		−0.108	−0.184	−0.050	**Supported**
i	Hum → Ctrl → Resist		−0.010	−0.046	0.022	Rejected
**H9**	**Favor → TPB → Misuse**					
a	Voc → Att → Misuse	0.122	−0.026	−0.171	0.112	Rejected
b	Voc → Norm → Misuse		−0.009	−0.144	0.141	Rejected
c	Voc → Ctrl → Misuse		0.086	0.011	0.185	**Supported**
d	Capt → Att → Misuse	0.853	−0.066	−0.185	0.050	Rejected
e	Capt → Norm → Misuse		−0.066	−0.194	0.053	Rejected
f	Capt → Ctrl → Misuse		0.068	−0.001	0.151	Rejected
g	Hum → Att → Misuse	0.000 **	0.091	−0.008	0.191	Rejected
h	Hum → Norm → Misuse		0.108	0.011	0.205	**Supported**
i	Hum → Ctrl → Misuse		0.062	0.004	0.135	**Supported**
**H10**	**Favor → TPB → Resist**					
a	Voc → Att → Resist	0.023 *	−0.013	−0.087	0.063	Rejected
b	Voc → Norm → Resist		−0.005	−0.082	0.070	Rejected
c	Voc → Ctrl → Resist		0.051	0.006	0.111	**Supported**
d	Capt → Att → Resist	0.582	−0.034	−0.104	0.026	Rejected
e	Capt → Norm → Resist		−0.038	−0.111	0.029	Rejected
f	Capt → Ctrl → Resist		0.036	−0.003	0.086	Rejected
g	Hum → Att → Resist	0.846	0.050	−0.001	0.115	Rejected
h	Hum → Norm → Resist		0.065	0.008	0.132	**Supported**
i	Hum → Ctrl → Resist		0.038	0.002	0.089	**Supported**

Note: * *p* < 0.05, ** *p* < 0.01. Note: LLCI and ULCI represent Bonferroni-adjusted 99.4444% percentile bootstrap confidence intervals (5000 bootstrap samples).

## Data Availability

The data are available from the corresponding author upon reasonable request.
